# Correction of Sagittal Balance With Resection of Kissing Spines

**DOI:** 10.7759/cureus.16874

**Published:** 2021-08-04

**Authors:** Eris Spirollari, Eric Feldstein, Christina Ng, Sima Vazquez, Merritt D Kinon, Chirag Gandhi, Rachana Tyagi

**Affiliations:** 1 Neurosurgery, Westchester Medical Center, Valhalla, USA

**Keywords:** kissing spines, baastrup’s disease, sagittal balance, sagittal imbalance, pelvic incidence

## Abstract

Kissing spines syndrome, also known as Baastrup’s disease, is a common yet underdiagnosed disorder involving close approximation of adjacent spinous processes. These painful pseudoarticulations may be secondary to the compensatory mechanisms that result from sagittal imbalance. Conventional operative correction of sagittal balance includes a wide range of procedures from facetectomies to vertebral column resection. Resection of kissing spines for the operative management of sagittal imbalance is a treatment modality not extensively discussed in the literature but may offer improved patient outcomes with shorter operative times, lower risk, and reduced length of stay.

A 67-year old male with a history of degenerative disk disease and scoliosis presented with neurogenic claudication and severe back pain that worsened with walking and improved with sitting. X-ray imaging of the lumbar spine revealed straightening of the normal lumbar lordotic curvature with mild rotoscoliosis. There was also evidence of retrolisthesis of L2 on L3 that worsened with flexion. The patient had Baastrup’s disease at the L3-4 and L4, 5 levels that contributed to his reduced range of motion on extension imaging. Operative treatments including long-segment fusion with interbody cages to correct sagittal balance were considered with a discussion of possible debilitating and high-risk post-surgical outcomes. Instead, the patient underwent a simple decompression surgery involving laminectomies and resection of kissing spines to correct his sagittal imbalance. Postoperative follow-up imaging demonstrated significant improvement in sagittal balance, and the patient expressed relief of back and leg pain.

Although underdiagnosed, consideration of kissing spines syndrome in the surgical correction of sagittal imbalance may offer an improvement over conventional operations. Our case presents a unique surgical perspective on the treatment of spinal stenosis with kissing spines with particular regard to correcting the sagittal imbalance, avoiding debilitating procedures, and providing better immediate postoperative outcomes.

## Introduction

Sagittal balance is a feature of the spine and body that maintains a mechanical balance in the sagittal plane. The purpose of this cone of equilibrium is to allow for a standing posture with minimal muscular energy expenditure. Coordination of spinal balance is accomplished through a relationship involving cervical lordosis, thoracic kyphosis, lumbar lordosis, and pelvic anatomical alignment [1]. In conditions that lead to progressive spinal malalignment, compensatory mechanisms utilize more energy expenditure and muscular effort to maintain an upright position. These spinal defects lead to intense muscular demand, pain, fatigue, and associated disability [2]. Sagittal imbalance is often found in cases of lower back pain.

Kissing spines syndrome, also known as Baastrup’s disease, is a relatively common but underdiagnosed disorder of the lumbar spine involving close approximation of adjacent spinous processes, causing pain with extension [3]. These most commonly occur at the level of L4-L5 and are secondary to degenerative processes and inflammation of the spine [4,5]. Clinical manifestations of kissing spine syndrome are similar to that of spinal stenosis. Midline distribution of back pain worsens with extension and is relieved with flexion. However, in contrast to spinal stenosis, kissing spine syndrome has midline tenderness that worsens on palpation and typically lacks neurogenic claudication [3]. Different treatment strategies exist depending on the severity of symptoms. For milder cases, conservative methods such as local steroid injections into the interspinous processes or oral analgesics can provide relief. For more severe cases of Baastrup’s disease, surgical treatment options include fusion of the lumbar spine or posterior spinous decompression [6].

Here, we present a 67-year-old man with a history of degenerative disk disease and scoliosis with severe back pain and neurogenic claudication attributed to spinal stenosis with kissing spine syndrome and sagittal imbalance. Rather than performing an extensive fusion and correction of the deformity, we opted to perform a simple decompression and resection of the kissing spines, to alleviate back pain and correct sagittal balance while avoiding debilitating post-surgical outcomes.

## Case presentation

An otherwise healthy and physically active 67-year-old man presented with severe lower back pain and bilateral leg pain more so in the right leg than left. His leg pain worsened with walking and improved with sitting consistently with neurogenic claudication. He also had diffuse tenderness to palpation along the midline of his lumbar back. At the time, the patient had been using a cane due to difficulty walking. He had a past surgical history of left ankle fusion with a leg-length discrepancy. Additionally, he was undergoing evaluation for bilateral knee replacements. Examination revealed the patient had a forward-leaning posture, bowed legs, pain with lumbar extension, and inability to stand on his toes and heels due to ankle fusion. His leg strength was normal and his sensation was grossly intact to light touch. X-ray imaging of the lumbar spine revealed straightening of the normal lumbar lordotic curvature with mild rotoscoliosis, but severe degenerative changes in the lumbar spine. The patient had Baastrup’s disease at the L3-4 and L4-5 levels that contributed to his reduced range of motion on extension imaging.

Standing preoperative sagittal scoliosis film revealed a coronal vertical axis of +51 mm and a sagittal vertical axis of +82 mm with the following pelvic parameters: 29 degrees of pelvic incidence (PI), 15 degrees of sacral slope (SS), and a pelvic tilt (PT) of 13 degrees. Further, there appeared to be a reversal of lumbar lordosis centered around L2-L3. Lumbar lordosis measured approximately -2 degrees from the superior endplate of L1 to the inferior endplate of L5. There was also a straightening of the thoracic kyphosis with approximately 26 degrees from the superior endplate of T1 to the inferior endplate of T12 (Figure 1).

**Figure 1:**
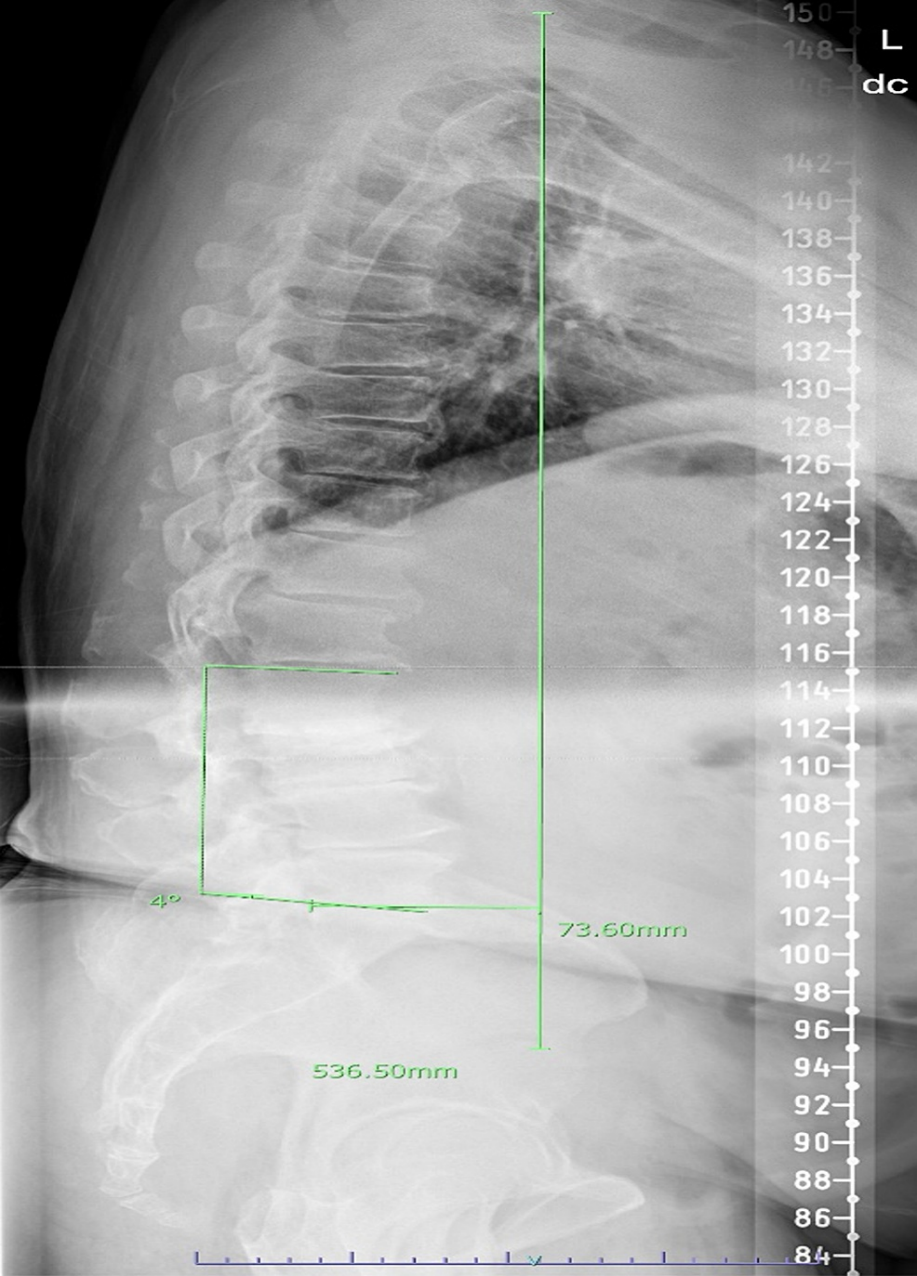
Preoperative sagittal scoliosis survey film showing forward sagittal balance, reversal of lumbar lordosis, and straightening of thoracic kyphosis.

MRI without contrast of the lumbar spine displayed multilevel degenerative disease with severe stenosis from L2-L5. Interestingly, his alignment changed when lying supine for the MRI and he appeared to regain some lumbar lordosis (Figures 2a-2e).

**Figure 2:**
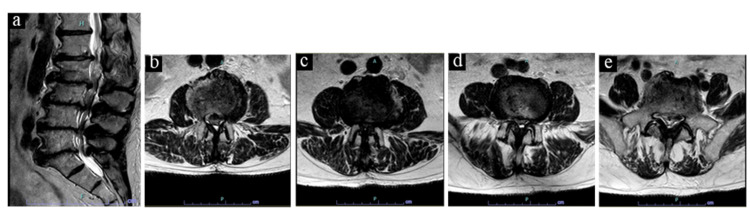
(a) Sagittal, (b) axial L2/3, (c) axial L3/4, (d) axial L4/5, and (e) axial L5/S1 non-contrast MRI images of lumbar spine preoperatively.

Intervention and outcome

Given the patient’s loss of lumbar lordosis and positive sagittal alignment, deformity correction surgery was considered; however, his symptoms were felt to be mostly neurogenic claudication related to his severe multilevel stenosis and less from his spinal deformity. We felt that his loss of lumbar lordosis was from his Baastrup’s disease at L3 and L4 since putting his lumbar spine into kyphosis increased the interspinous distance. Open full decompressive lumbar laminectomies with bilateral medial facetectomies and foraminotomies from L3-L5 were recommended in order to address his multilevel stenosis and resect his spinous processes causing Baastrup’s disease.

The patient tolerated the procedure well and was discharged home on the same day. The postoperative course was uncomplicated and involved physical therapy to work on core strengthening exercises to improve his overall spinal alignment.

On follow-up, the patient has a resolution of his back pain and radicular leg pain. Likewise, he reported feeling “much straighter” while walking as he no longer had pain when leaning backward. Follow-up standing scoliosis survey films two months and six months postoperatively demonstrated significantly improved sagittal forward balance and improvement in both his lumbar lordosis and thoracic kyphosis (Figures 3a, 3b), as well as improvement in his pelvic parameters (Table 1).

**Figure 3:**
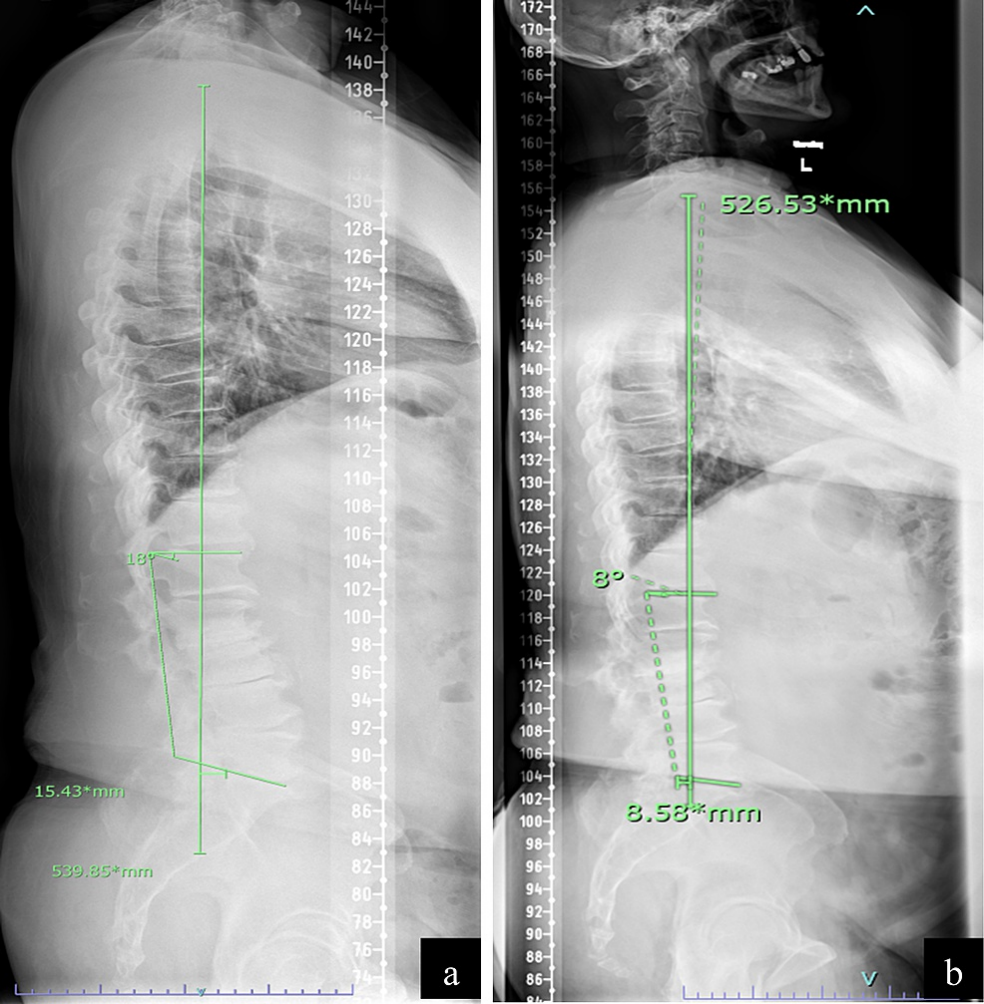
(a) Two-month and (b) six-month postoperative sagittal scoliosis survey film demonstrating improved sagittal forward balance, correction of lumbar lordosis, and decreased thoracic kyphosis.

**Table 1:** Preoperative and postoperative spine measurements

	Preoperative Measurements	Two-Month Postoperative Measurements	Six-Month Postoperative Measurements
Lumbar Lordosis	-2 degrees	13 degrees	8 degrees
Pelvic Incidence (PI)	29 degrees	31 degrees	34 degrees
PI-LL Mismatch	31 degrees	18 degrees	26 degrees
Sacral Slope	15 degrees	17 degrees	20 degrees
Pelvic Tilt (PT)	13 degrees	12 degrees	15 degrees
Sagittal Vertical Axis	82 mm	-18 mm	9 mm

## Discussion

Kissing spines are frequent radiological findings but their significance may not be fully understood, which could lead to less effective treatment [7]. Particularly, the likely contribution to sagittal imbalance as part of the disease process is addressed by our case. A recent study by Maes et al. found an overall incidence of Baastrup’s disease of 8.2% in a population of 539. Moreover, the study reported associations between the presence of kissing spines with older age, central canal stenosis, disc bulging, and anterolisthesis [8]. Treatment of the disease is an ongoing topic of debate. While most of the literature relevant to the management of the disease suggests surgical interventions or intraspinous injections, a past cohort study of 64 patients of ages ranging from 21 to 74 with a peak in the 40 to 70 years age group by Beks et al. [9] found that partial or total surgical excision of the lumbar spinous processes does not always alleviate pain [7,9]. Our patient’s case offers a unique surgical perspective on the treatment of spinal stenosis with kissing spines, particularly with the implications it has in spinal sagittal deformity. We obtained a positive outcome with correction of pelvic parameters and associated symptoms with decompressive surgery alone.

Studies continue to demonstrate the value in addressing sagittal plane deformity for pain control, improvement of function, and overall quality of life [1,10]. Likewise, the management of spinal deformities is often directly correlated to maintaining or restoring individual sagittal plane alignment to ensure proper physiologic distribution of mechanical stresses and to limit further complications [11]. Past studies have demonstrated that 74% of age-related disc degeneration is due to genetic factors and is associated with disc mechanical incompetence, bone remodeling, and hypertrophy of the articular facets in the lumbar spine. These deformities contribute to lumbar hypolordosis and kyphosis, which are thought to be the primary causes of anterior sagittal imbalance [12,13]. While lumbar kyphosis is directly correlated to degenerative spinal variations of aging, modifications of other spinopelvic parameters relate to the compensatory mechanisms that worsen sagittal imbalance. These mechanisms act to maintain the upright position with minimal muscular effort [13].

Traditional non-operative methods in the management and correction of sagittal imbalance include bracing for structural support or physical therapy for strengthening. Operative treatment includes a wide range of procedures to restore the alignment of the spine, ranging from facetectomies to vertebral column resection using various combinations of approaches [1]. A retrospective cohort study by Champagne et al. compares the three widely used lumbar interbody fusion approaches and found that only oblique lumbar interbody fusion (OLIF) managed to significantly improve segmental and lumbar lordosis. Additionally, all approaches, including open transforaminal lumbar interbody fusion (TLIF), minimally invasive TLIF (MIS TLIF), and OLIF were found to significantly augment disk height, with OLIF producing the greatest effect. These results suggest that compared to open and MIS TLIF, OLIF may offer the most effective correction of sagittal balance [14]. However, the authors did not evaluate patients for Baastrup’s disease. Some of the sagittal balance correction may have been obtained by increased distraction on the posterior elements, thereby increasing the interspinous distance, allowing improved lordosis.

Further studies have shown the importance of optimizing quality control in the reconstruction of sagittal balance by not only correctly orienting the pelvis through the restoration of L1-S1 lordosis with adequate distribution at L4-S1 segments but also through persistent rehabilitation of surgically injured lumbar extensor musculature and prevention of junctional fractures [15]. To promote optimal postoperative outcomes, an understanding of whole spinal and spinopelvic alignment and dynamics is necessary [16]. However, there remains an inadequate body of evidence on the consideration of Baastrup’s disease in the operative correction of sagittal imbalance.

Kissing spines is thought to result from the compensatory mechanisms that limit the consequences of lumbar kyphosis from degenerative disk disease shifting the axis of gravity anteriorly. In response, sacral slope decreases, PT increases, and thoracic kyphosis may decrease. These changes increase the stress on the posterior elements of the lumbar spine with resultant facet hypertrophy. Thus, Baastrup’s disease may result from interspinous hyper pressure caused by compensatory lumbar extension with the loading of the posterior elements [17]. As a result, this may paradoxically worsen sagittal balance by reducing the ability to extend.

In older patients, the potential presence of hip-spine syndrome, described as concurrent pathology at both the hip and spine, is an important consideration that impacts treatment decisions. Offierski and MacNab distinguish three categories of hip-spine syndrome: simple (either hip or the spine as the single source of symptomatic disability), complex (overlap in symptoms from both sources), and secondary (inter-related pathology). Fixed flexion of the hip can rotate the pelvis forward and lead to lumbar hyperlordosis, lower back pain, and foraminal stenosis [18]. Fixed adduction of the hip can lead to pelvic misalignment and secondary lumbar scoliosis [18]. Advanced osteoarthritis and decreased range of movement at the hip joint are also associated with increased spinal sagittal misalignment, which can contribute to lower back pain [19]. When deciding treatment strategies, an investigation into the true source of symptoms allows surgeons to target the areas which will most likely relieve patient concerns [20].

Various surgical treatments were considered for our patient. Lumbar spinal fusion was discussed, but anterior or lateral fusion with a lordotic cage likely would have had limited success in correcting the kyphosis due to the presence of the extremely hypertrophic spinous processes limiting extension. We also feared that by doing an anterior correction without removing the spinous processes at the affected areas, we would further irritate Baastrup’s disease by increasing the lumbar lordosis and decreasing the interspinous distance. Most of his symptoms were related to his neurogenic claudication from his stenosis and since the patient’s alignment improved when supine and not weight-bearing, we felt his spine was flexible enough to hopefully restore alignment through postoperative core strengthening once his pain was improved. The patient underwent a spinous decompression surgery involving resection of kissing spines. Postoperative follow-up imaging demonstrated significant improvement in sagittal balance, and the patient expressed relief of both back and leg pain. This case demonstrates the favorable outcomes of surgical resection of kissing spines for the treatment of lower back pain and sagittal imbalance without the need for extensive deformity correction.

## Conclusions

In cases of progressive spinal malalignment, compensatory mechanisms develop to maintain the upright position. These compensatory mechanisms attempt to correct for sagittal imbalance; however, they are maladaptive and have negative consequences such as disability involving intense muscular demand, pain, and fatigue. Kissing spines is thought to result from increased stress on the posterior structures secondary to compensatory lumbar extension. Recognition of kissing spines as a common contributor of sagittal imbalance allows for avoidance of debilitating procedures and provides better immediate outcomes for patients. Our case offers a unique surgical perspective on the treatment of spinal stenosis with kissing spines with particular regard to correcting the sagittal balance. We demonstrate successful treatment of sagittal plane imbalance and lumbar stenosis via resection of kissing spines, suggesting favorable outcomes can be obtained with this focused surgical approach.
